# Non-traumatic Tension Faecopneumothorax: A Rare Complication of Intrathoracic Colonic Perforation

**DOI:** 10.7759/cureus.97816

**Published:** 2025-11-25

**Authors:** Ana Tojal, Ines Pinto Pereira, Catarina Pacheco, Érico Costa, Sara A Pinto, Ana P Oliveira

**Affiliations:** 1 Intensive Care Unit, Unidade Local de Saúde Gaia/Espinho, Vila Nova de Gaia, PRT; 2 Intensive Care Unit, Unidade Local de Saúde Gaia/Espinho, Vila Nova De Gaia, PRT

**Keywords:** diaphragmatic hernia, intensive care unit, intestinal perforation, pneumothorax, thoracic surgery

## Abstract

Tension faecopneumothorax is an exceptionally rare and life-threatening condition, typically resulting from bowel herniation through the diaphragm with intrathoracic perforation. Although diaphragmatic hernias can be congenital, traumatic, or iatrogenic, colonic herniation and subsequent perforation into the thoracic cavity are exceedingly uncommon and diagnostically challenging. We report the case of a 53-year-old woman with a history of type 2 diabetes mellitus and a recent laparoscopic cholecystectomy, admitted to the emergency department (ED) with severe respiratory distress. Despite multiple prior visits to the ED with nonspecific symptoms, this time the patient presented in shock. Chest radiography revealed a left-sided tension pneumothorax, and insertion of a chest tube resulted in the unexpected drainage of feculent material. Computed tomography confirmed a left hydropneumothorax secondary to central diaphragmatic hernia with intrathoracic colonic perforation. Emergency surgery included segmental colectomy with colostomy, diaphragmatic repair, and chest tube placement. Postoperatively, the patient was admitted to the intensive care unit with septic shock. Despite subsequent improvement, a secondary empyema necessitated repeat drainage. Persistent infection and loss of lung viability led to left lower lobectomy. After 65 days of hospitalization and a multidisciplinary approach involving intensivists and surgical teams, the patient was discharged with a favorable clinical evolution. This case highlights the diagnostic complexity and severity of tension faecopneumothorax due to colonic perforation in a diaphragmatic hernia, particularly in the absence of trauma. It underscores the importance of considering atypical etiologies in patients with unexplained respiratory distress and the need for prompt imaging, surgical intervention, and comprehensive intensive care. Given the rarity of such presentations, reporting these cases contributes to improved clinical awareness and management strategies.

## Introduction

Diaphragmatic hernia can occur congenitally or be acquired, with the latter often associated with trauma or iatrogenic causes. Herniation of abdominal contents through the diaphragm can lead to severe complications, such as bowel obstruction and perforation, which are rare and challenging to diagnose and manage [1,2]. The presence of fecal matter and air in the pleural cavity, known as faecopneumothorax, is an unusual event that occurs when a diaphragmatic defect allows intra-abdominal contents to herniate into the thoracic cavity, combined with a tear or laceration in the colon resulting in a colopleural fistula. Tension faecopneumothorax is one of the uncommon but potentially fatal causes of severe respiratory failure [3,4]. A literature search in PubMed using the following mesh terms was conducted: hydropneumothorax; pneumothorax; hernia, diaphragmatic, traumatic; hernia, hiatal; hernias, diaphragmatic, congenital (Query: ("Hydropneumothorax"[MeSH Terms] OR "Pneumothorax"[MeSH Terms] OR "Hernia, Diaphragmatic, Traumatic"[MeSH Terms] OR "Hernia, Hiatal"[MeSH Terms] OR "Hernias, Diaphragmatic, Congenital"[MeSH Terms]). Only 14 cases of faecopneumothorax were found in the literature [3-16], seven of which presented as tension pneumothorax [3,4,11-15]. This case report aims to highlight the rarity and complexity of managing tension faecopneumothorax secondary to colonic perforation.

## Case presentation

A 53-year-old woman with a history of type 2 diabetes mellitus, depressive syndrome, and cholecystectomy for gallstone disease (five months before), was admitted to the emergency department with severe respiratory distress. After three visits to the emergency service the previous week, she had been diagnosed with intercostal neuritis and treated with symptomatic medication without clinical improvement. On admission, she presented with left-sided chest and hypochondrial pain (6/10 in Visual Analog Scale), without relief or aggravating factors or irradiation. There were no associated symptoms. Physical examination revealed a patient in poor general condition, with severe respiratory distress characterized by oxygen saturation of 78% on room air, tachypnea, and use of supraclavicular accessory muscles. Breath sounds were markedly diminished over the left lung field. Vital signs showed a heart rate of 190 bpm, blood pressure of 90/63 mmHg, and clinical signs of poor peripheral perfusion. A tension pneumothorax was suspected, and a chest tube (18Fr) was inserted in the left fifth intercostal space at the midaxillary line. Drainage of feculent material was observed, raising concern for intrathoracic bowel perforation.

Due to persistent respiratory distress, the patient underwent endotracheal intubation for ventilatory support. Arterial blood gas analysis demonstrated high anion gap metabolic acidosis with hyperlactatemia (Table 1). In addition, laboratory studies revealed acute kidney injury (Kidney Disease: Improving Global Outcomes (KDIGO) stage 2) and elevated inflammatory markers.

**Table 1:** Relevant analytic study FiO_2 _= Fraction of inspired oxygen.

Arterial Blood Gas (FiO_2_ 0.5, 37ºC)		Reference	Laboratory Study		Reference
pH	7.1	7.35-7.45	Hemoglobin (g/dL)	15.2	13 – 18
pCO_2_ (mmHg)	56.7	35 - 45	Leucocytes (x10^3^/uL)	16.58	3.8 – 10.6
pO_2_ (mmHg)	74.6	80-100	Platelet count (x x10^3^/uL)	329	140 - 440
HCO_3_^- ^(mmol/L)	17.7	22-26	Creatinine (mg/dL)	1.49	0.67 – 1.17
Base excess (mmol/L)	-12.2	-2 - +2	Urea (mg/dL)	84	17 - 50
Anion Gap (mmol/L)	20.1	8 – 12	Myoglobin (ng/mL)	191	28 - 72
Glucose (mg/dL)	453	70-110	Troponin T hs (ng/L)	13	< 14
Lactate (mmol/L)	6.8	0.5 - 2	Reactive Protein C (mg/dL)	55.12	0 – 0.5

**Figure 1:**
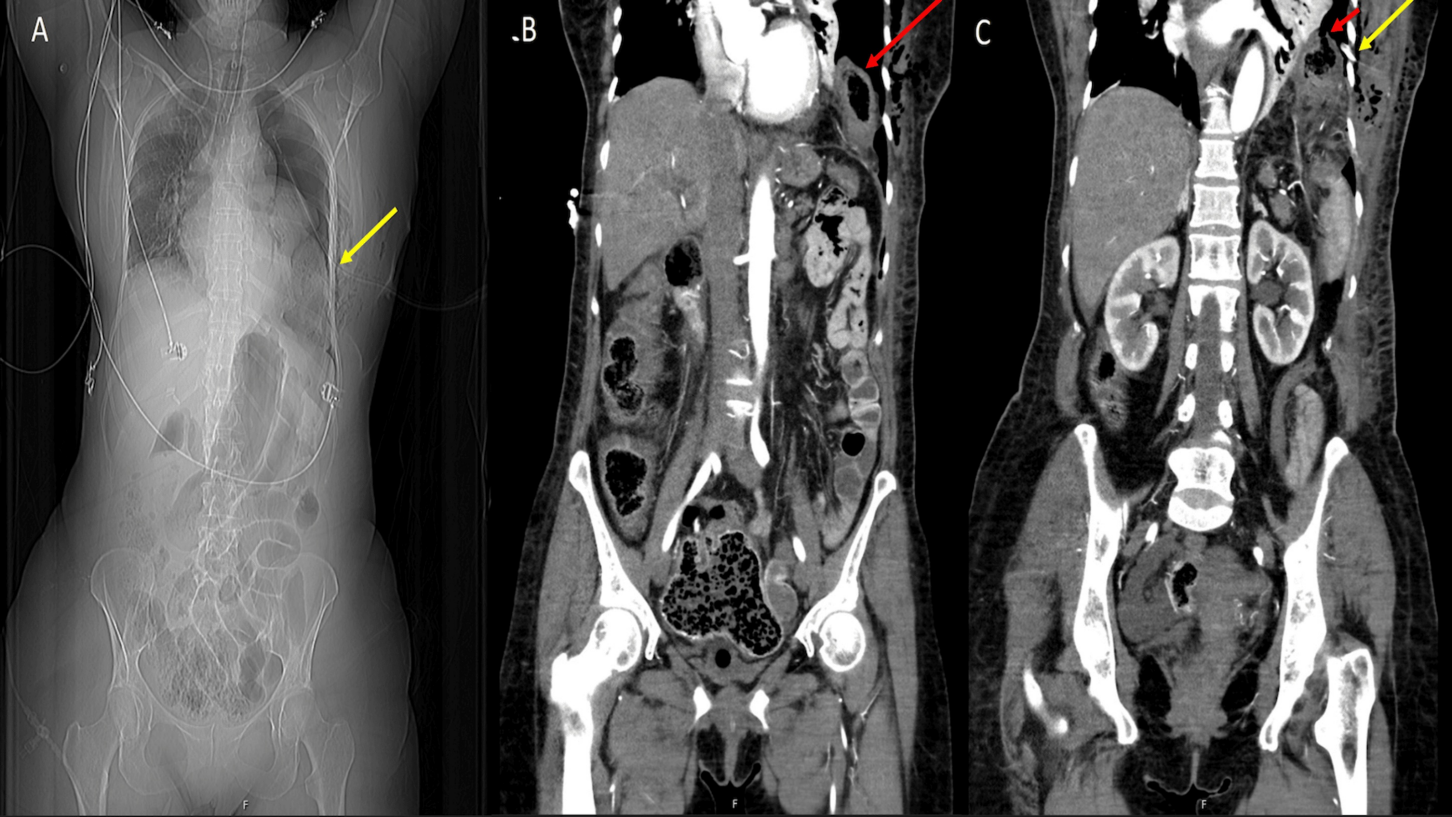
Contrast-enhanced thoraco-abdomino-pelvic CT images Contrast-enhanced thoraco-abdomino-pelvic computed tomography demonstrating the presence of the colon in the left hemithorax through a central diaphragmatic defect. Panel A - Topogram; Yellow arrow pointing to the left chest tube. Panel B - Coronal plane with red arrow pointing to the intrathoracic colon. Panel C – Coronal plane with red arrow pointing to the intrathoracic perforated colon and yellow arrow demonstrating the presence of a left chest tube.

Contrast-enhanced computed tomography (CECT) of the thorax and abdomen confirmed left hydropneumothorax, with central diaphragmatic herniation and colonic perforation (Figure 1). Emergency surgery was performed with reduction of the herniated content, left segmental colectomy, colostomy creation, diaphragmatic defect repair, and insertion of a new left chest tube.

**Figure 2:**
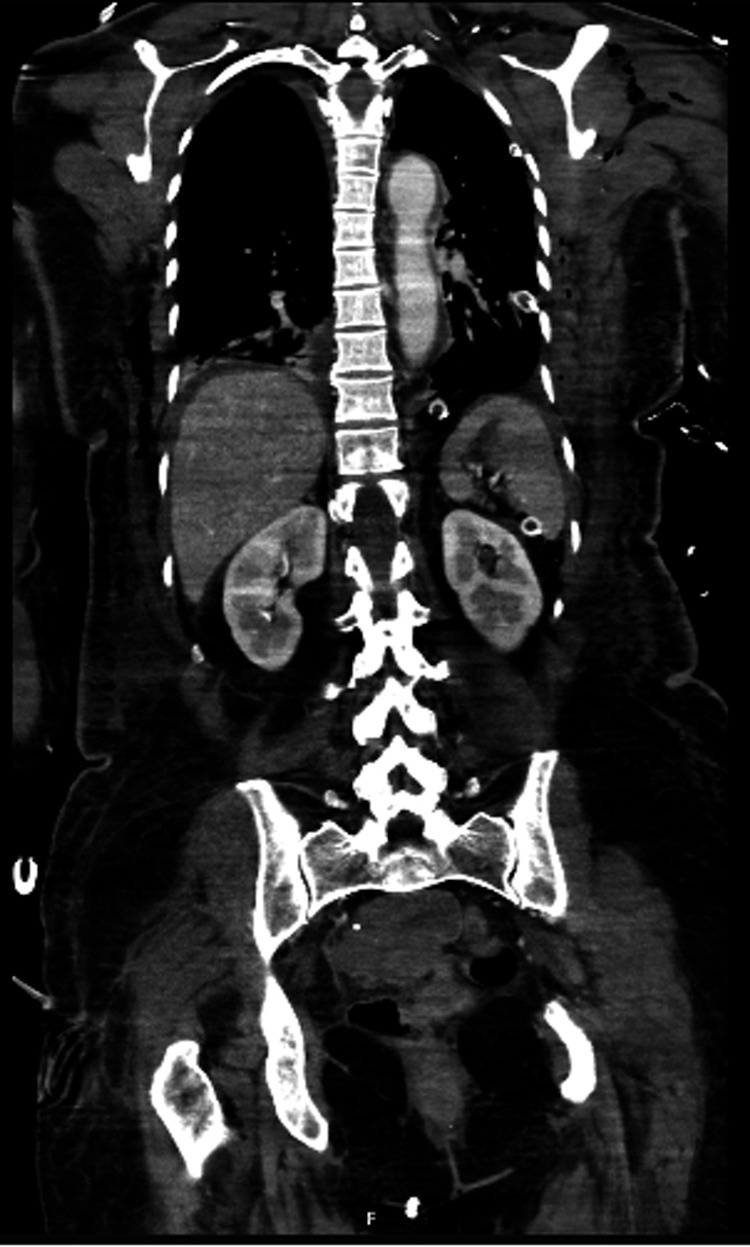
Post-operative non-contrast thoraco-abdomino-pelvic computed tomography

The patient developed septic shock, requiring admission to the Intensive Care Unit (ICU), under empirical antibiotic therapy with ceftriaxone 1 g every 12 hours and metronidazole 500 mg every eight hours. The following day, a non-contrast computed tomography (CT) confirmed the correct position of the chest tube and excluded complications (Figure 2). 

**Figure 3:**
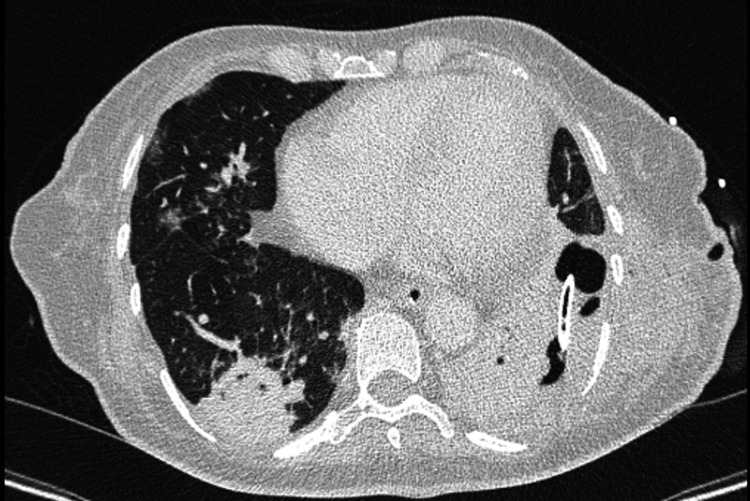
Reassessment thoracic computed tomography Atelectasis with consolidation of the left lower lobe and consolidation involving part of the right lower lobe.

In the first 72 hours of hospitalization, there was a favorable clinical evolution, with resolution of septic shock originating from faecopneumothorax and fecaloid peritonitis. On the ninth day of hospitalization, due to persistent fever and rising inflammatory markers, a chest CT scan was performed, revealing a left loculated pleural effusion. A diagnostic thoracentesis confirmed empyema (pH 6.7, lactate 12 mmol/L), and a new chest drain was inserted. Concurrently, a ventilator-associated pneumonia (VAP) was diagnosed, prompting escalation of antibiotic therapy to piperacillin/tazobactam 4.5 g every six hours. Bronchoalveolar lavage cultures yielded *Stenotrophomonas maltophilia*, which was assumed to be the causative pathogen of the VAP, and trimethoprim-sulfamethoxazole was initiated. Empyema fluid cultures grew *Escherichia coli*, *Stenotrophomonas maltophilia*, and *Candida parapsilosis*. Based on the antimicrobial susceptibility profiles, piperacillin/tazobactam and trimethoprim-sulfamethoxazole were continued, and fluconazole was added to target *Candida parapsilosis*, which was confirmed to be susceptible. Despite targeted antimicrobial therapy, the clinical picture persisted. This, in conjunction with recurrent atelectasis (as shown in reassessment thoracic CT, Figure 3) and lack of functional recovery, led to a multidisciplinary discussion between the intensive care and thoracic surgery teams. A decision was made to proceed with surgical intervention, which was performed on day 30 of hospitalization. During the procedure, pleural drainage and left lower lobectomy were carried out due to the non-viability of this lung segment.

The postoperative course was complicated by a high-output bronchial fistula with subcutaneous emphysema. Considering the successful weaning from mechanical ventilation and discontinuation of positive pressure support, the patient did not require surgical intervention. The patient received 44 days of antibiotic therapy. It is noteworthy that during the ICU stay, the patient developed intubation-associated pneumonia, without a significant clinical impact on the evolution of the primary infectious condition. The patient showed a slow but favorable clinical evolution and was discharged after 65 days of hospitalization. Twelve months after the event, the patient had already undergone surgery for restoration of colonic continuity (colostomy reversal) and showed no functional sequelae after an intensive rehabilitation program.

## Discussion

Congenital hernias can be subdivided into two subtypes based on anatomical location: posterolateral (Bochdalek hernia), the most common, and anterior (Morgagni hernia) [9,17]. Although rare, congenital hernias can present clinically only in adulthood [1,18]. Traumatic diaphragmatic hernias are rare, more frequent on the left side, result from blunt or penetrating abdominal trauma, and can be asymptomatic (incidentally discovered on imaging exams) or have a wide spectrum of clinical presentations [9,12,19]. They may present with nonspecific symptoms such as dyspnea and chest pain or abdominal complaints, including abdominal pain, nausea, vomiting, or signs of bowel obstruction, depending on the involved abdominal organ [20]. When abdominal viscera herniate into the thorax, signs of respiratory difficulty are common [8]. These hernias can manifest acutely or present years after the initial injury [5,8,11]. Tension pneumothorax is more commonly caused by trauma, particularly penetrating trauma, and is very rarely spontaneous (typically in divers and newborns) [2,3]. 

Iatrogenic diaphragmatic hernias have been described following laparoscopic cholecystectomy, hepatectomy, splenectomy, gastrectomy, as well as other abdominal surgeries [2,21]. This patient underwent a laparoscopic cholecystectomy five months before this acute event, with no reference to abdominal or thoracic trauma. There was no mention or clinical evidence of a diaphragmatic hernia in previous imaging exams, suggesting postoperative iatrogenesis as a possible etiological factor.

When comparing this case with previously reported cases in the literature, several key aspects emerge regarding management strategies and patient outcomes. Surgical intervention, most frequently involving laparotomy, with or without thoracotomy, is consistently described as essential in the treatment of complicated diaphragmatic hernias with visceral perforation. Despite similar surgical approaches, outcomes vary widely, while some patients recovered fully without sequelae [8,11,15,22], others experienced significant morbidity or mortality, particularly in the presence of bowel ischemia or septic shock [4,6,7,9,13]. Although rarer and less frequently described than post-traumatic cases, iatrogenic diaphragmatic hernias have also been reported [6,7,9,14], with variable clinical outcomes. Postoperative complications are frequent and significant, occurring in up to 43.5% of patients, mainly due to pulmonary issues such as empyema and prolonged mechanical ventilation [3,4]. In this case, the prolonged ICU stay, need for surgical reintervention, and extended rehabilitation illustrate the complex clinical course often associated with delayed diagnosis and secondary complications. These findings underscore the importance of early recognition, prompt surgical management, and anticipation of potential postoperative complications.

## Conclusions

This case underscores the clinical severity and complexity of fecopneumothorax, a rare entity that can arise not only from blunt trauma but also as a complication of intra-abdominal procedures. Communication between abdominal and pleural compartments can lead to a life-threatening emergency. The atypical presentation and rapid clinical deterioration highlight the need for a high index of suspicion in patients with recent abdominal surgery or unexplained respiratory symptoms. Primary survey with stabilization of airway, breathing, and circulation is of utmost priority, followed by prompt chest decompression and definitive surgical management.

The protracted clinical course, marked by multiple infectious complications and the need for surgical reintervention, reinforces the importance of a comprehensive and patient-centered strategy. A multidisciplinary team approach was essential to navigate the critical phases of treatment and achieve full functional recovery following an intensive rehabilitation process. Beyond its rarity, this case contributes to existing literature by underscoring the necessity of sustained postoperative vigilance and multidisciplinary follow-up to optimize outcomes and minimize morbidity in such complex presentations.
